# Percutaneous internal fixation with Y-STRUT® device to prevent both osteoporotic and pathological hip fractures: a prospective pilot study

**DOI:** 10.1186/s13018-017-0514-2

**Published:** 2017-02-09

**Authors:** François H. Cornelis, Lambros Tselikas, Thibault Carteret, Bruno Lapuyade, Thierry De Baere, Jean Charles Le Huec, Frédéric Deschamps

**Affiliations:** 1 Pellegrin Hospital, CHU Bordeaux, Bordeaux, France; 2 Tenon Hospital, Radiology Department, APHP, Paris, France; 3 Gustave Roussy Cancer Campus Grand Paris, Villejuif, France; 4 Haut-Leveque Hospital, CHU Bordeaux, Bordeaux, France

**Keywords:** Pathological hip fracture, Osteoporosis, Bone metastases, Prophylactic consolidation, Proximal femur, Biomechanical reinforcement

## Abstract

**Background:**

We studied Y-STRUT® (Hyprevention, France), a new percutaneous internal fixation device, in combination with bone cementoplasty to prevent hip fracture.

**Methods:**

Between February 2013 and February 2015, a total of 16 femoral necks in 4 osteoporotic and 12 oncologic patients have been considered for prophylactic consolidation in this prospective multicentre pilot study involving 4 different hospitals. These consolidations were performed percutaneously under fluoroscopic guidance using Y-STRUT®, a dedicated internal fixation device. For osteoporotic patients, orthopaedic surgeons performed the prophylactic consolidations immediately after surgical treatment of a hip fracture (same anaesthesia) in the opposite side. For oncologic patients, without current hip fracture but considered at risk (Mirels score ≥8), interventional radiologists performed the procedures. We report the preliminary results of feasibility, safety and tolerance of these preventive consolidations using Y-STRUT®.

**Results:**

Four patients (mean 83 years old) had prophylactic consolidation because of a severe osteoporosis (mean *T*-score −3.30) resulting in first hip fractures. Ten patients (mean 61 years old) were treated because of impending pathological fractures (mean Mirels score 9) related to femoral neck osteolytic metastases. All the procedures were performed with success. Wound healing was achieved in all cases with no access site complication. Radiographic exams performed at 3 months follow-up revealed that Y-STRUT® was well integrated in the bone. For the osteoporotic cohort, mean pain was 0.9 ± 0.7 at 3 weeks. For the oncologic cohort, it decreases from 3.6 ± 2.9 at baseline to 2.4 ± 0.9 at 2 months.

**Conclusions:**

Preliminary results demonstrate the feasibility and safety of Y-STRUT® implantation as well as the tolerance of the device.

## Background

Hip fracture may occur in several situations, such as low-energy trauma, osteoporosis or tumour invasion. Hip fracture is a public health issue due to a large and growing incidence and its functional and vital repercussions. The worldwide number of hip fractures is estimated at 2 million in 2010, a 26% increase since 1990 [1], and could reach 6.3 million in 2050 [2].

In patients suffering from osteoporosis, this event often remains highly disabling despite the effectiveness of surgical treatments and the mortality rate after a first hip fracture is between 15 and 30% [3]. Moreover, in 20% of cases, this first fracture is followed by a second fracture of the opposite hip (contralateral) within 5 years (10% during the first year, 15% at 2 years). These patients, greatly reduced physically, see their mortality risk increase to up to 64% in the following 5 years [4]. Hip fracture is, in most cases, the result of a fall linked to a daily living activity associated with age-related bone degeneration or osteoporosis. Osteoporotic elderly women are the most concerned by hip fracture. Solutions are currently proposed for the prevention of hip fracture related to osteoporosis. They consist in hip protectors (preventive treatment with immediate effect) and osteoporosis drug treatments [5] (long-term preventive treatment). However, these solutions, ineffective in practice mainly due to lack of patient adherence to treatment, are not satisfactory. Biomechanical investigations were performed on some prophylactic surgical techniques [6, 7], but to our knowledge, only one was the object of a clinical trial [8] and is not marketed.

In patients with metastatic cancer, bone metastases, mostly localized at the trochanteric region and femoral neck [9], cause pathological fractures of particularly serious consequences for these patients, are highly vulnerable and are difficult to operate. Life expectancy of patients who have suffered a fracture of the proximal femur is estimated to be less than a year on average [10]. In order to prevent pathological fractures due to bone metastases, only few options currently exist. A metallic osteosynthesis fixation device can be used in some cases [11], presenting the advantage of being very robust to sustain possible rapid lesion growth, but it is an invasive procedure at risk for this patient profile, making more complex further radiation therapy and requiring temporary chemotherapy cessation. Other techniques are reported in the literature such as triple screwing combined to cementoplasty [12, 13] or femoroplasty with or without needle prior insertion [14]. However, the products used in these techniques are not indicated for prophylactic fixation, and these attempts are not standardized methods.

An alternative implantable device, Y-STRUT® (Hyprevention), has been developed in these indications. It is a medical device that is to be implanted during a minimally invasive procedure in the proximal femur in order to enhance its biomechanical performance and prevent hip fracture.

A multicentre, single-arm, prospective study was initiated in order to assess the Y-STRUT® medical device in an orthopaedics-traumatology indication—prevention of contralateral hip fracture following a first pertrochanteric fracture—and in an oncologic indication—impeding pathological fracture prevention due to metastatic lesion located in the neck or trochanter.

## Methods

### Ethical considerations

All procedures were performed according to the Declaration of Helsinki, and the Human Ethics Committee of the concerned countries approved all procedures used. Initial clinical investigation plans and substantial amendments were reviewed by ethics committees as requested by the French and Belgian regulations. Initial protocols and substantial changes were authorized by the Competent Authorities from the participating Member States (ANSM (Agence Nationale de Sécurité du Médicament et des produits de santé) in France and AFMPS (Agence Fédérale des Médicaments et des Produits de Santé) in Belgium).

### Inclusion criteria

#### Osteoporotic cohort

A total of 15 patients were planned to be included in this cohort. The patients were recruited on arrival to the emergency room following a low-energy trauma (fall from the patient’s height, fall from the bed, missed stair steps, etc.) leading to a pertrochanteric fracture. The main inclusion criteria were sex (only women selected), age (between 60 and 90 years old) and osteoporosis (suspicious or known and defined by a *T*-score inferior or equal to −2.5). The patients underwent the surgical procedure within 24 to 72 h following their admission.

#### Oncologic cohort

A total of 10 procedures were planned. The patients were selected during multidisciplinary consultation meetings between oncologists, orthopaedic surgeons and interventional radiologists. The main inclusion criteria were patients with lytic lesions located in the proximal femur, with a size inferior or equal to two thirds of the cortex, and having a high risk of pathological hip fracture defined by a Mirels score superior or equal to 8 [15]. Once included, the patients had the procedure planned and performed in the interventional radiology department within the two following weeks.

### Investigational device description

**Fig. 1:**
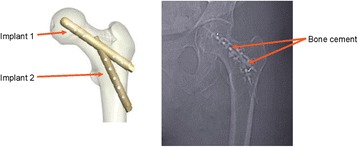
Y-STRUT® implants (schematic and x-ray views)

The Y-STRUT® device was designed to provide a prophylactic reinforcement of the proximal femur. It is an alternative implantable device which consists of two implants made of a radiotransparent PEEK polymer material. These implants connect in situ (Fig. 1) and work in combination with polymethyl methacrylate (PMMA)-type bone cement. Perforated implants allow controlled injection of the cement (see Fig. 1). The cement aims to increase the surface contact between the bone and the device and to anchor the device in the proximal femur. It is also used to fill the lytic lesions, if any.

The consolidation of the femoral head by the implantable medical device was studied through biomechanical tests in vitro, on human femurs, in order to simulate falls on anatomical pieces. This study demonstrated the potential of Y-STRUT® to improve the biomechanical performance of the proximal femur. Implant insertion seems to be relevant to support multiple falls and, thus, to prevent a second hip fracture in elderly patients. As the studied device is not intended to be used as a fracture fixation osteosynthesis system, but as a preventive reinforcement in case of osteoporosis or localized osteolytic lesion, its mechanical performance was not designed to be compared to existing fracture fixation devices (short femoral nail or compression hip screw).

### Operative technique

**Fig. 2:**
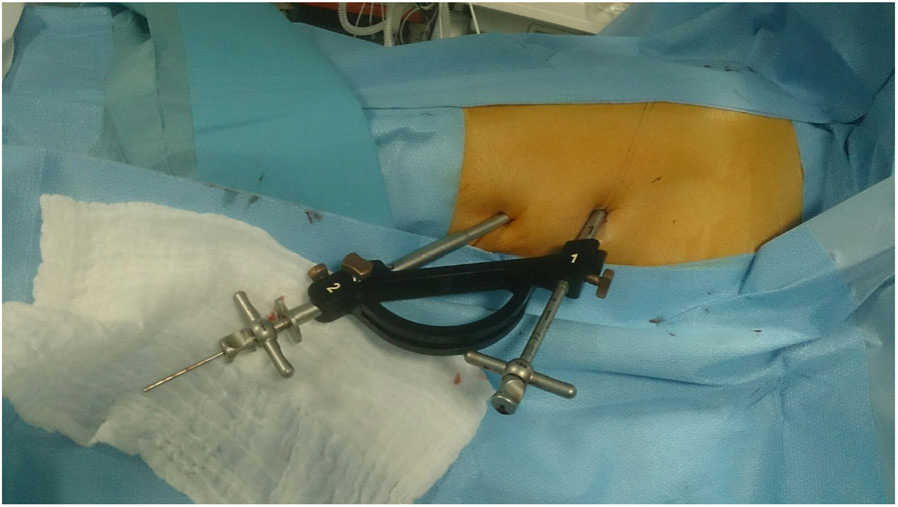
Percutaneous approach

The two implants composing the device are inserted through a minimally invasive procedure under imaging control. The dedicated instrumentation ensures the connection between the two components with a minimally invasive approach (Fig. 2). The technique consists in introducing a guide wire in the axis of the femoral neck to direct the drilling for the implant 1. A pilot, on which work tubes are assembled, enables the guidance of the second drill to connect the 2 components of the device. The device is finally fixed into bone with PMMA cement injected through the implants.

In this study, all procedures were performed under general anaesthesia.

#### Specifications for osteoporotic cohort

Prophylactic consolidation with the implantable medical device was performed contralaterally by orthopaedic surgeons, after surgical treatment of the pertrochanteric fracture on the opposite side and under the same anaesthesia. The patients were in a supine position on the orthopaedic traction table. A bioactive PMMA radio-opaque bone cement at low temperature of polymerization (CORTOSS®, Stryker) was used.

#### Specifications for oncologic cohort

**Fig. 3:**
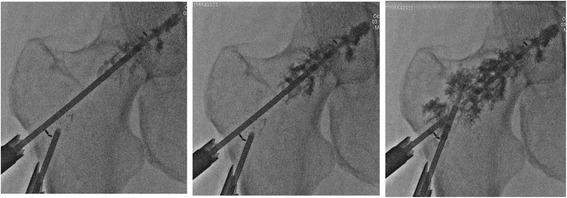
Radiotransparent implant has been implemented. The bone metastasis is being filled with PMMA bone cement

All oncologic procedures were performed by interventional radiologists in this indication, with a similar operative technique and identical instrumentation kit. The patients were either in a supine position or in a lateral position maintained by a scoop stretcher. Lytic femoral lesions of the patients were filled while cement was injected through the implant at the last stage of the procedure (Fig. 3). The patients received OPACITY+® cement (TEKNIMED) or F20® cement (TEKNIMED). Both are PMMA radio-opaque bone cements at high temperature of polymerization.

### Post-operative protocol and follow-up

No specific rehabilitation was needed. Full weight bearing on the Y-STRUT® implanted leg and walking recovery were allowed the day following the intervention. The patients from the osteoporotic cohort had to follow the rehabilitation protocol prescribed for their fracture. A 5-year-long follow-up is planned, with short-term (3 weeks, 3 months and 6 months), mid-term (12 months) and long-term (2, 3, 4 and 5 years) assessments for the osteoporotic cohort. A 1-year-long follow-up is planned for the oncologic cohort, with short-term (2 and 6 months) and mid-term (12 months) assessments.

Patients were followed by medical consultation and radiographic exams.

### Data analysis

The investigators committed to follow the protocols in all respects particularly in regard to obtaining consent, following patients and reporting serious adverse events. All investigation variables were collected using electronic Case Report Forms (CS Online, Clinsight), and a quality control of the database was done to confirm the overall integrity of the data. Adverse events, medical history and associated treatments were coded using the MedDRA and the WHO Drug Dictionaries. A questionnaire to be completed by the physicians performing the interventions was used to assess the technical feasibility of each operative step.

#### Endpoints

Common and primary objectives of these two investigations were to assess the procedure feasibility and safety as well as the short-term and mid-term tolerance of Y-STRUT®.

Evaluation of the procedure feasibility and safety was based on procedural parameters: technical difficulties (on a scale from 1 to 5), patient’s positioning, operating time (skin to skin), hospitalization stay and quantity of cement, and on intraoperative adverse events, including cement leakage, implantation failure or any other event likely to impact the benefit of the procedure and/or to present a risk for the patient.

Six short- and medium-term tolerance device criteria were defined: walking recovery at short-term (and plantar pressure for the osteoporotic cohort), pain evaluation using a visual analogue scale (VAS) at each follow-up visit, overall patients’ condition assessment using Oxford Hip Score (OHS-12), osteointegration and stability of the device by radiographic control, adverse events possibly related to the device or its implantation and device explantation.

Secondary endpoints focused on the mid-term efficacy and included low-energy falls and fractures reported during post-operative follow-up.

### Statistical and quantitative assessments

The statistical analysis was performed using SAS version 9.3 (SAS Institute, NC, USA). Quantitative variables were described using mean, standard deviation (SD), median and range. Qualitative variables were described using frequencies and percentages of each modality. Number of patients and number of missing data were given for each variable.

## Results

### Patients

Four patients out of a total of 15 planned were implanted in the osteoporotic cohort. Patient inclusion is still ongoing. The patients, all female per protocol, were on average 83 years old (range 81 to 86) and in a severe osteoporotic condition, as confirmed by a mean *T*-score of −3.30 (range −3.8 to −2.7) measured at 3 months. The 10-year probability of hip fracture was calculated for each patient at discharge by using the FRAX® tool (Fracture Risk Assessment Tool). Results indicated a mean risk of 22% (range 10 to 37).

Twelve patients were included in the oncologic cohort. Patients (8 males, 4 females) were all somewhat ambulatory (with a mean ECOG (Eastern Cooperative Oncology Group) score of 1.4; range 0 to 3) and presented with impending pathologic fracture at baseline (as indicated by a median Mirels score of 9 (range 8 to 11)). Primary cancers were lung (*n* = 5), kidney (*n* = 2) or a variety of other primary cancers (*n* = 5). It is to be noted that 2 patients experienced femoral fracture prior to the intervention; in accordance with the protocol, they were not implanted; they were excluded from post-operative results.

### Technical feasibility

Results showed that all procedural steps were found either “easy” or “very easy” in most cases, demonstrating the technical feasibility of the procedure despite the novelty of the intervention and the limited physician experience (each of the 8 treating physicians performed less than 2 interventions on average).

In the osteoporotic cohort, all the procedures were performed by orthopaedic surgeons, in a supine position on the orthopaedic traction table. The mean volume of cement injected was 7.3 ml (range 6 to 10 ml), and the mean duration of the intervention was 48 ± 15 min (range 35 to 65 min) (Table 1).

**Table 1:** Technical data and outcomes—osteoporotic cohort

Patient	Procedure duration (min)	Hospitalization duration (days)	Pain (VAS) at 3 weeks
1-1	35	5	2
1-2	35	19	0
1-3	58	17	0.5
1-4	65	27	1

In the oncologic cohort, all the procedures were performed by interventional radiologists. The patients were in a supine position (50%) or in a lateral position (50%) on an angiographic table, in an angio-suite. The mean volume of cement injected was 9.2 ml (range 3 to 15 ml), and the mean duration of intervention was 97 ± 28 min (range 60 to 155 min) (Table 2).

**Table 2:** Technical data and outcomes—oncologic cohort

ID	Procedure duration (min)	Hospitalization duration (days)	Pain (VAS) at baseline	Pain (VAS) at discharge	Pain (VAS) at 2 months
2-1	75	24^b^	9	NA	NA
2-2^a^	NA	NA	NA	NA	NA
2-3	60	2	2	2	2
2-4	132	1	3	0	3
2-5	155	3	3	3	3
2-6	91	1	0	4	3
2-7	105	5	8	8	Missing
2-8^a^	NA	NA	NA	NA	NA
2-9	90	1	2	Missing	3
2-10	75	3	Missing	Missing	1
2-11	110	4	4	6	3
2-12	85	1	1	1	1
Mean	97	2.3	3.6	3.4	2.4

*NA* Not applicable

^a^Cancelled due to pre-operative fracture

^b^Patient deceased while she was still hospitalized. Excluded from statistical calculation of the hospitalization duration

There was no case of wound infection, bleeding, leakage or inflammation reported.

### Outcomes

#### Osteoporotic patients

Average follow-up duration was 461 days (range 213 to 945 days).

Duration of hospitalization was not lengthened by the additional implantation. The mean duration was 17.0 days (range 5 to 25 days). All patients had recovered their walking ability at discharge. At 3 weeks and 3, 6 and 12 months, comparison between the two legs’ plantar pressures revealed no differences.

The mean pain was 0.9 (range 0.5 to 2) at 3 weeks (Table 1). A high pain was reported for 1 patient, due to a cement leakage. Pain was resolved by the removal of the excess cement. Evolution of OHS-12 score was reported for a single patient with an OHS-12 of 18 at 3 months (severe condition) and of 43 at 2 years (satisfactory condition). Wound healing was achieved for all patients. No osteolysis nor implant loosening was observed at the different follow-ups. No fracture occurred among this cohort.

#### Oncologic patients

During the follow-up, 6 patients (60%) deceased from severe progression of their underlying cancer after a mean follow-up of 142 days (range 24 to 324 days)—there was no other cause of death reported. Among the survival patients, the average follow-up duration was 305 days (range 246 to 393 days).

The mean duration of hospitalization was 2.3 days (range 1 to 5 days—1 patient suffered from a severe progression of his cancer and deceased 24 days after the implantation while he was still hospitalized; he was excluded from this statistical calculation), and 4 of the 10 patients (40%) were discharged the day following the intervention.

No cement leakage was observed. Wound healing was achieved in all cases with no access site complication.

Eight out of 9 patients (89%) could resume walking after the intervention. The patient who did not recover walking was the only one presenting an ECOG score of 3 before intervention (meaning more than 50% of time in bed during the day and symptomatic).

The average pain at baseline was 3.6 ± 2.9, with no evolution at discharge with an average pain of 3.4 ± 2.6. However, at 2 months, pain relief was observed with an average pain of 2.4 ± 0.9 (Table 2). One patient complained from pain at the level of the implant entry site in the bone immediately after the procedure. The pain was spontaneously resolved at 3 weeks follow-up. No significant change was observed in OHS-12 results at baseline, 2 months and 6 months (average total score of 30, 28 and 32, respectively).

One patient was diagnosed at 6 months follow-up with an asymptomatic femoral neck fracture associated with a fracture of the implant. This event may be attributed to tumour progression and non-optimal placement of the Y-STRUT® implant (non-compliant with the instructions for use), too close to the superior cervical cortex which might weaken the bone. The patient underwent orthopaedic surgery on the same day to explant the device and treat the fracture, without further related complications.

## Discussion

The technical feasibility was demonstrated with a favourable assessment achieved in both cohorts. Minimally invasive procedure is a real need in prophylaxis to avoid any risk of infection and to get rapid recovery, as well for osteoporotic patients who already undergo a more invasive fixation fracture intervention with the inherent risks, as for oncologic patients with poor performance status and life expectancy and who are not candidates for a surgery. In both indications, no bleedings nor infection was related to the Y-STRUT® implantation.

Y-STRUT® has the advantage of being specifically developed for prophylactic fixation, as shown through the biomechanical evaluation [16]. It is made of polymer, thus allowing an adequate stress distribution into the bone to achieve reinforcement. To our knowledge, osteosynthesis devices are neither indicated nor used for prophylaxis in osteoporotic patients. They are made of titanium or stainless steel that make them strong enough to fix a fracture and could be used to reinforce the bone, but they are invasive, may be painful for a prophylactic fixation and may require a longer hospitalization than Y-STRUT® [11]. However, in oncology, in the case of large lesions affecting the diaphysis, intramedullary nails are still indicated. Y-STRUT® is only indicated in the case of small lesions located in the trochanter or the femoral neck when the implant placement will allow reinforcement.

The mean duration of intervention, skin to skin, was 48 ± 15 min when performed by orthopaedic surgeons. The lengthening of the anaesthetic for the concerned patients (additional to the one needed for the fracture treatment for pre-operative patient preparation and device implantation) did not lead to any adverse events. In oncology, the feasibility of the Y-STRUT® implantation by interventional radiologists was shown. The mean duration was 97 ± 28 min, slightly shorter than the 110 ± 43 min reported by Deschamps et al. [13] in their cohort of 35 patients. Tian et al. [14] reported a little shorter duration with a mean of 80 ± 7.5 min in a cohort of 19 patients, for the insertion of 4 modified trocar needles combined with cementoplasty. Compared to these techniques, the Y-STRUT® implantation has the advantage to be based on a dedicated instrumentation kit which ensures the reproducibility of the technique. The longest implantation duration for interventional radiologists can be explained by the time-consuming CT scans performed during the procedure. Moreover, procedures for both indications were performed by 9 different practitioners, and duration should be significantly improved with a learning curve. Over time, implantation duration should not be longer than the one for a standard osteosynthesis device.

As expected, the total quantity of cement injected in Y-STRUT® was lower in the osteoporotic patients than in the oncologic patients (7.3 ± 1.9 ml versus 9.2 ± 3.1 ml, respectively). Indeed, in oncology, where the cement was also used to fill the lytic lesion, the quantity of cement injected (mean 9.2 ± 3.1 ml) increased significantly with the Mirels score, which takes the size of the lesion into account. These figures appear relatively low when compared to the volumes of 36 and 47 ml injected alone by Heini et al. [6] and Sutter et al. [7], respectively, to reinforce osteoporotic bones by femoroplasty in cadaveric studies and to the volume of 31 ml injected by Tian et al. [14] in metastatic bone lesions treated by internal fixation with needles plus cementoplasty. It is similar to the volume of 6 ml (range 3 to 10 ml) injected during triple screwing combined with cementoplasty [13] for the same indication but with 20% of cement leakage reported compared to only 1 case (7%) with Y-STRUT® in this study. Moreover, the limited amount of cement injected when implanting Y-STRUT® provides favourable conditions if a future explantation and revision are necessary.

In the osteoporotic cohort, the average duration of hospitalization was 17.0 ± 9.1 days. It was related to the hip fracture treatment and was not lengthened by the Y-STRUT® implantation. For the oncologic cohort, the average hospitalization was 2.3 ± 1.4 days (range 1 to 5 days), a little shorter than the duration of 3.1 days (range 1 to 8 days) reported in a cohort of 35 oncologic patients after triple screwing fixation [13]. These durations appear very short when compared to the 19.3 days (range 1 to 105 days) reported by Ristevski et al. [11] in a cohort of 201 patients for prophylactic treatment of femoral metastatic lesions with a fracture fixation system such as intramedullary nailing, showing the interest of a minimally invasive technique. In addition, it should be noted that 4 of the 10 patients from the study in the oncologic indication (40%) were discharged the day following the intervention, suggesting that the Y-STRUT® implantation could be performed as a day-surgery procedure.

For osteoporotic patients, average pain at the Y-STRUT®-implanted hip was 0.9 (range 0.5 to 2) at 3 weeks follow-up, indicating a very good short-term tolerance of the device. More patients are needed to assess the mid-term pain evolution and must show that prophylaxis does not lead to any local pain. All patients could resume walking after implantation with a good balance. The amount of missing data for OHS-12 scores does not allow to draw conclusions on the overall patients’ outcome. However, no fracture was recorded after a mean follow-up of 461 days (range 213 to 945 days).

In oncologic patients, overall patients’ condition, evaluated with OHS-12, remains stable (average total score of 30, 28 and 32 at baseline, 2 months and 6 months, respectively). All patients presenting an ECOG score inferior to 3 at baseline (89%) could resume walking after the intervention. A mean pain relief from 3.9 ± 2.9 at baseline to 2.4 ± 0.9 at 2 months was observed. Two patients pre-included in the oncologic cohort experienced femoral fracture prior to the intervention; in accordance with the protocol, they were neither included nor treated and therefore not followed. However, these pre-operative fractures confirm the validity of the eligibility criteria and illustrate the severity of the patients’ condition in this indication. In addition, 1 case of post-operative fracture at 6 months was reported for an oncologic patient that may be attributed to tumour progression and non-compliant placement of the Y-STRUT® implant. Similarly, triple screwing plus cementoplasty treatment technique [13] led to 2 cases (6%) of post-operative pathological fractures at 3 weeks and 7 months. However, with cementoplasty alone, a higher fracture rate (24%) was reported in a cohort of 21 patients [14], which tends to show the lower efficacy of this technique.

Limitations of the study include a small patient sample (14 patients implanted with the studied device) and a short follow-up (mean follow-up is 383 days among the survival patients when the cohorts are combined). Nevertheless, the presented data is sufficient to evaluate the feasibility of the procedure. Additional studies should be conducted on a greater number of patients and with a longer follow-up to confirm the clinical benefits of the Y-STRUT® implantation in both indications.

## Conclusions

Preliminary results from these first-in-man studies demonstrate the feasibility, safety and tolerance of the Y-STRUT® implantation. This procedure is associated with a dedicated material and a controlled operative technique, compared to existing non-standardised percutaneous techniques. Y-STRUT® represents a promising consolidation technique in two clinical indications: (1) for patients suffering from bone fragility, it addresses a common issue with a new specific approach easy to implement, with immediate effect contrary to existing treatments; the procedure can be performed during the same anaesthetic than the fracture fixation one without delaying the patient recovery, and (2) for oncologic patients, whom general status is often a contra-indication to a prophylactic consolidation with a more invasive osteosynthesis fixation system, this percutaneous treatment is an interesting alternative for localized lytic lesion of the proximal femur.

## Abbreviations

AFMPS: Agence Fédérale des Médicaments et des Produits de Santé (Belgium health authorities)
ANSM: Agence Nationale de Sécurité du Médicament et des produits de santé (French health authorities)
ECOG: Eastern Cooperative Oncology Group
OHS: Oxford Hip Score
VAS: Visual analogue scale
